# IL-6 secreted by cancer-associated fibroblasts promotes epithelial-mesenchymal transition and metastasis of gastric cancer via JAK2/STAT3 signaling pathway

**DOI:** 10.18632/oncotarget.15119

**Published:** 2017-02-06

**Authors:** Xiongyan Wu, Pan Tao, Quan Zhou, Jie Li, Zhenjia Yu, Xiaofeng Wang, Jiaanfang Li, Chen Li, Min Yan, Zhenggang Zhu, Bingya Liu, Liping Su

**Affiliations:** ^1^ Department of Surgery, Shanghai Key Laboratory of Gastric Neoplasms, Shanghai Institute of Digestive Surgery, Ruijin Hospital, School of Medicine, Shanghai Jiao Tong University, Shanghai 200025, People's Republic of China

**Keywords:** cancer-associated fibroblasts, interleukin-6, JAK/STAT3, gastric cancer

## Abstract

Cancer-associated fibroblasts (CAFs), as the activated fibroblasts in tumor stroma, are important modifiers of tumor progression. However, the molecular mechanisms underlying the tumor-promoting properties of CAFs in gastric cancer remain unclear. Here, we show that CAFs isolated from gastric cancer produce significant amounts of interleukin-6 (IL-6). CAFs enhances the migration and EMT of gastric cancer cells through the secretion of IL-6 that activates Janus kinase 2/signal transducers and activators of transcription (JAK2/STAT3) pathway in gastric cancer cells, while deprivation of IL-6 using a neutralizing antibody or inhibition of JAK/STAT3 pathway with specific inhibitor AG490 markedly attenuates these phenotypes in gastric cancer cells induced by CAFs. Moreover, silencing IL-6 expression in CAFs or inhibiting JAK2/STAT3 pathway in gastric cancer cells impairs tumor peritoneal metastasis induced by CAFs *in vivo*. Taken together, these results suggest that CAFs in the tumor microenvironment promote the progression of gastric cancer through IL-6/JAK2/STAT3 signaling, and IL-6 targeted therapy could be a complementary approach against gastric cancer by exerting their action on stromal fibroblasts.

## INTRODUCTION

Gastric cancer, one of the most common primary malignant tumors, is the third leading cause of cancer death in worldwide with the highest estimated mortality rates in Eastern Asia [1]. Despite advances in early detection, diagnosis, and treatment of gastric cancer, the overall prognosis is still poor and the 5-year survival for patients with gastric cancer has remained 20–25%, which is due to the recurrence and metastasis after surgery [2]. Thus, a better understanding of the molecular mechanisms in promoting the progression of gastric cancer will be helpful to improve the prevention, diagnosis, and treatment of gastric cancer.

Epithelial-mesenchymal transition (EMT), a well-characterized embryological process, has been identified to play a critical role in tumor progression, including invasion and metastasis, by which cancer cells could gain more aggressive properties. In the process of EMT, epithelial cells undergo a phenotypic switch by lossing their cell polarity and the epithelial markers (E-cadherin, β-catenin), to form mesenchymal cells through acquiring the mesenchymal markers (N-cadherin, vimentin, ZEB2), thus these transformed epithelial cells acquire fibroblast-like properties and exhibit reduced cell-cell adhesion and increased motility [3–5]. The enhanced motility and invasiveness afforded by EMT is critical in the initiation of metastasis for cancer progression, and the acquisition of a mesenchymal phenotype has been also enhanced resistance to chemotherapy and poor prognosis [6, 7]. The expression of these EMT markers can be induced by a number of growth factors/cytokines such as hepatocyte growth factor (HGF), transforming growth factor (TGF)-β, CXCL12 and hypoxia-inducible factor-1α (HIF-1α) [8–11].

Accumulating evidence has indicated that interactions between tumor and stromal cells create a unique microenvironment that is essential for tumor growth, invasion, and metastasis [12, 13]. Therefore, epithelial cell-stromal cell interactions usually act as the regulators of EMT, and the factors inciting EMTs are often originated from the stromal cells creating the tumor microenvironment. Cancer-associated fibroblasts (CAFs), the important components of tumor stroma, are the key players in regulating tumor progression [12, 13]. It has been observed that CAFs actively communicate with cancer cells through growth factors or inflammatory cytokines such as HGF, IL-6, TGF-β, VEGF, FGF, and CXCL12 that can promote tumorigenesis and progression [12–16]. IL-6 is a multifunctional cytokine that was originally determined to be a regulator of immune and inflammatory responses [17] and has been also reported to be associated with certain epithelial tumors such as colon cancer, prostate cancer [18, 19]. Aberrant production and signaling of IL-6 is tightly linked to tumor generation and poor disease outcome in many cancer types, including gastric cancer. IL-6 exerts its effects by binding to IL-6α chain and a common cytokine receptor signal-transducing subunit gp130, which leads to activation of the Janus kinases (JAKs) family of tyrosine kinases and the signal transducers and activators of transcription (STAT) family, particularly STAT3 [20]. Activation of IL-6-JAK-STAT3 signaling pathway plays an active role in the oncogenesis of a variety of tumors [21–23]. Previous studies have demonstrated that STAT3 is activated in gastric cancer tissues. Menheniott TR, et al. detected STAT3 in antral biopsies, and have demonstrated that both total STAT3 and phosphorylated STAT3 increased in intestinal-type gastric cancer compared with normal stomach. Zhang XM et al also found that activated STAT3 is positive in early gastric cancer, poorly differentiated adenocarcinoma and metastatic lymph node tissues [24, 25]. However, the role of CAFs and IL-6 in gastric cancer has not been well addressed. Therefore, our aim was to determine how CAFs can enhance tumor metastasis and EMT changes of gastric cancer cells, and link CAFs with activation of the IL-6-JAK-STAT3 signaling pathway in the progression of gastric cancer.

In this study, we find that CAFs isolated from gastric cancer produce considerable amounts of IL-6. CAFs-derived IL-6 enhances the migration and EMT of gastric cancer cells by the activation of JAK2/STAT3 pathway in gastric cancer cells, while deprivation of IL-6 using a neutralizing antibody or inhibition of JAK2/STAT3 pathway with specific inhibitor AG490 markedly reduces these phenotypes in gastric cancer cells induced by CAFs. Moreover, knockdown the expression of IL-6 in CAFs by RNAi or inhibiting JAK2/STAT3 pathway in gastric cancer cells by the specific inhibitor AG490 significantly retards the tumor peritoneal metastasis induced by CAFs *in vivo*. These results suggest that suppressing IL-6 or its downstream targets could serve as an effective therapeutic strategy against gastric cancer by exerting their action on stromal fibroblasts.

## RESULTS

### IL-6 is highly expressed in CAFs in the tumor microenvironment of gastric cancer

**Figure 1:**
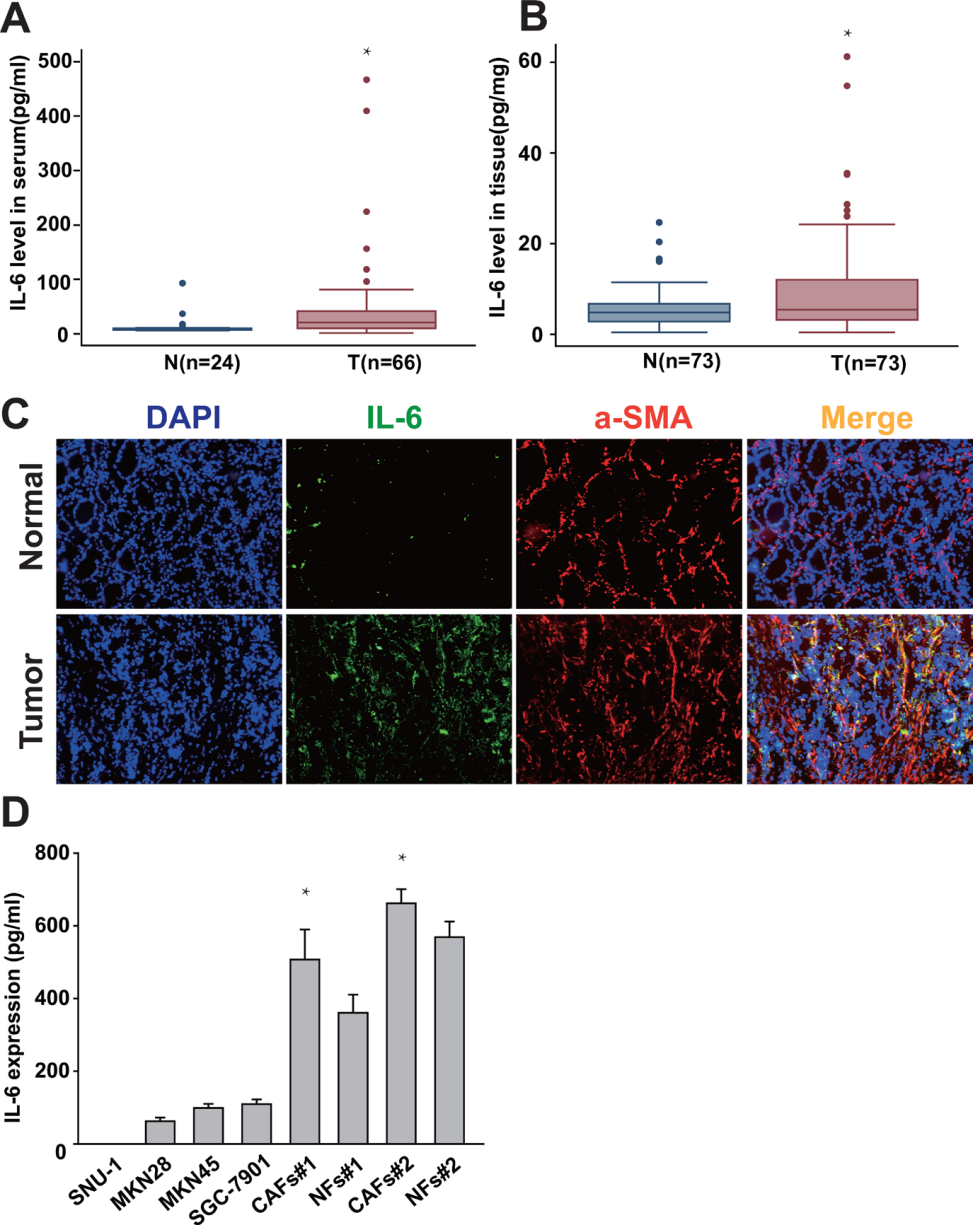
IL-6 is highly expressed in gastric CAFs (**A**, **B**) The expression of IL-6 in both serum and cancer tissues in gastric cancer patient was detected by ELISA. (**C**) Frozen tissue sections from gastric cancer and adjacent non-tumor tissue were immunostained by DAPI (nucleus), FITC (IL-6) and PE (α-SMA) (200×). (**D**) IL-6 protein expression level in the gastric cancer cells (SNU-1, MKN45, SGC7901 and MKN28), gastric CAFs and normal fibroblasts (NFs) was quantified 24hrs after change the culture medium by ELISA. *P < 0.05.

Cancer-associated stroma secretes a plethora of factors such as HGF, IL-6, TGF-β to promote the growth and invasion of the underlying tumor [16]. It has been reported that IL-6 is associated with certain epithelial tumors. To determine the expression level and cellular source of IL-6 in gastric cancer, we first detected IL-6 expression in both serum and cancer tissues in gastric cancer patient by ELISA. As shown in Figure 1A, IL-6 levels in the serum of gastric cancer patients were significantly elevated in comparison with healthy volunteers. Similarly, the expression level of IL-6 was also significantly higher in the cancer tissues compared with adjacent non-cancerous tissues (Figure 1B). To further illustrate which cell component(s) in gastric cancer tissues is (are) responsible for the high expression of IL-6, we detected IL-6 expression in tissues by immunofluorescence staining. As shown in Figure 1C, the expression of IL-6 was noted in stromal cells of peri- and intra-tumoral areas rather than in the cytoplasm or the nucleus of the cancer cells. Anti α-SMA antibody are used to identify CAFs. Upon immunofluorescence staining, we identified both α-SMA and IL-6 expressing stromal cells in peri- and intra-tumoral areas (Figure 1C). We further quantified expression levels of IL-6 in CAFs, normal fibroblasts (NFs) and gastric cancer cells (SNU-1, MKN45, SGC7901 and MKN28) by ELISA. We found IL-6 level were significantly higher in CAFs compared to NFs and gastric cancer cells (Figure 1D). These findings suggest that IL-6 is overexpressed in gastric cancer and CAFs is one major cell source in producing IL-6 in the tumor microenvironment of gastric cancer.

### CAFs enhance the migration of gastric cancer cells via the secretion of IL-6

**Figure 2:**
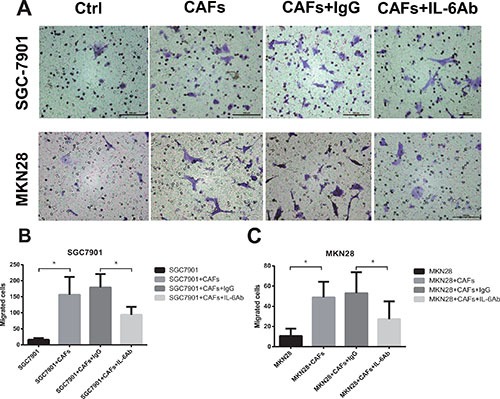
CAFs enhance the migration of gastric cancer cells via the secretion of IL-6 (**A**) The effect of CAFs on cell migration was determined 24 hrs after in the presence of IL-6 neutralizing antibody or IgG isotype control antibody. Representative photographs of migratory cells on the membrane (magnification, 100×) are shown. (**B**, **C**) Migratory Cells were counted in ten randomly selected microscopic fields. Values are represented as mean ± SD of three independent experiments. *P < 0.05.

We examined the migration of gastric cancer cells induced by CAFs, a key determinant of metastasis in tumor progression. As shown in Figure 2A and 2B, SGC-7901 cells co-cultured with CAFs showed enhanced ability of migration than SGC-7901 cells alone (SGC-7901 cells alone, 15.8 ± 5.0 cells per field; SGC-7901 cells co-cultured with CAFs, 156.4 ± 55.5 cells per field; *P* < 0.01). However, adding neutralizing IL-6 antibody into the co-culture system led to significantly decreased migration (neutralizing IL-6 group, 93.6 ± 24.9 cells per field; isotype control group, 179.0 ± 41.9 cells per field; *P* < 0.01) of SGC-7901 cells. Similarly, MKN28 cells co-cultured with CAFs also exhibited higher ability of migration than MKN28 cells alone (MKN28 cells alone, 10.6 ± 7.3 cells per field; MKN28 cells co-cultured with CAFs, 48.8 ± 15.4 cells per field; *P* < 0.01), while the migratory ability of MKN28 cells co-cultured with CAFs was significantly reduced by the addition of anti-IL-6 neutralizing antibody (neutralizing IL-6 group, 27.2 ± 17.7 cells per field; isotype control group, 53.0 ± 20.8 cells per field; *P* < 0.01) (Figure 2A and 2C). Moreover, we determined the migration ability of gastric cancer cells induced by exogenous IL-6. Gastric cancer cells stimulated by IL-6 showed enhanced ability of migration compared with gastric cancer cells alone (Supplementary Figure 1). Thus, these data suggest that CAFs enhance the migration of gastric cancer cells via the secretion of IL-6.

### CAFs promote EMT changes of gastric cancer cells via the secretion of IL-6

**Figure 3:**
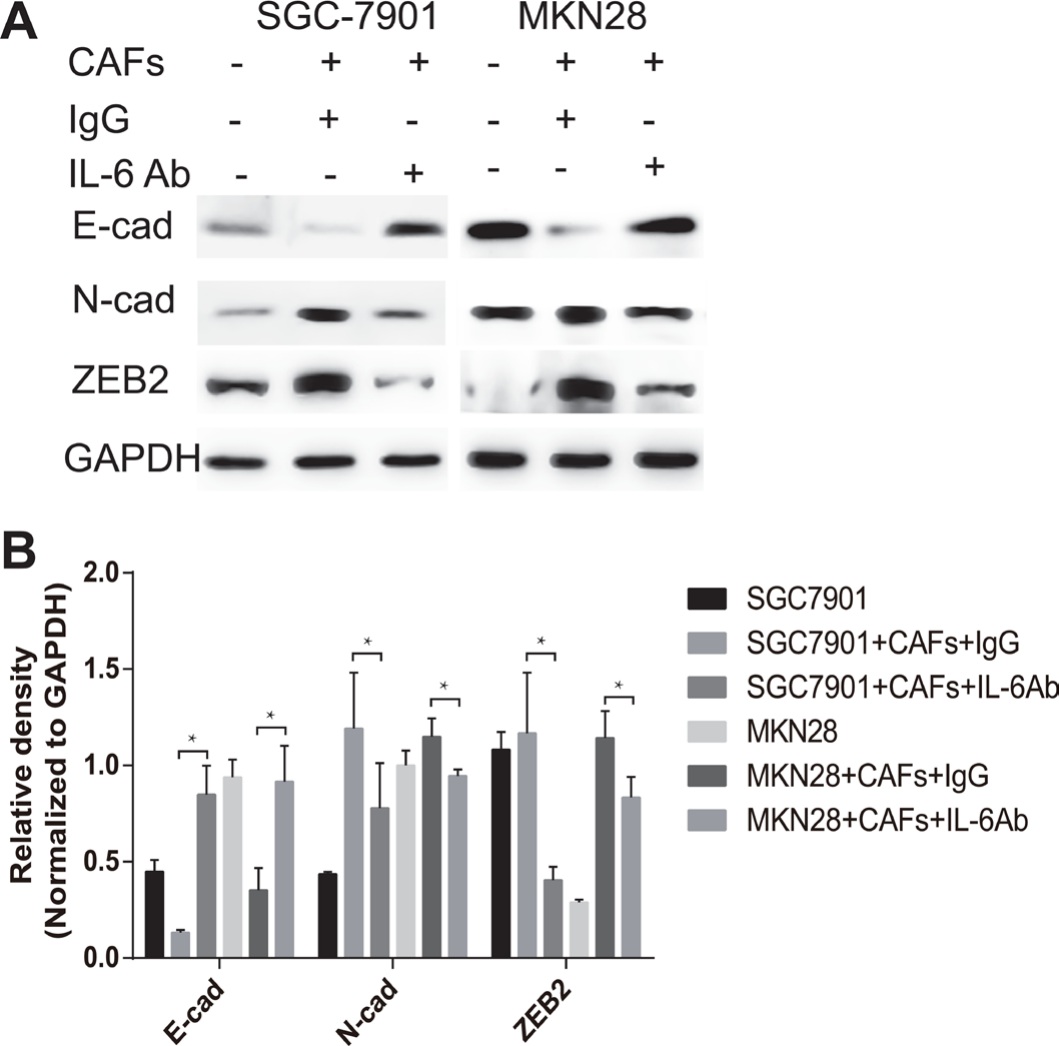
CAFs promote EMT of gastric cancer cells via the secretion of IL-6 (**A**) Protein expression of E-cadherin, N-cadherin and ZEB2 in gastric cancer cells SGC-7901 and MKN28 co-cultured with CAFs in the presence of IL-6 neutralizing antibody or IgG isotype control antibody was analyzed by western blot. Representative images from one of the three independent experiments are presented. (**B**) Densitometric analysis of E-cadherin, N-cadherin and ZEB2 expression is shown.

EMT, a well-characterized embryological process, has been identified to play a critical role in tumor metastasis, which is characterized by losing epithelial markers (*e.g*. E-cadherin), and acquiring of mesenchymal markers (*e.g*. N-cadherin, ZEB2) [5]. To examine the role of CAFs in mediating EMT in gastric cancer cells, we cultured SGC-7901 cells or MKN28 cells with CAFs in a previous described co-culture system [14]. As shown in Figure 3A and 3B, co-culture of CAFs with SGC-7901 cells markedly decreased the expression of E-cadherin and simultaneously increased the expression of N-cadherin and ZEB2 in SGC-7901 cells. The similar phenotype changes of EMT were also occurred in MKN28 cells after co-cultured with CAFs (Figure 3A and 3B). To determine whether IL-6 contributes to the EMT effect of CAFs on gastric cancer cells, we added the IL-6 neutralizing antibody into the co-culture system. As shown in Figure 3A and 3B, EMT induced by CAFs was impaired when the IL-6 was abolished by its specific neutralizing antibody, which was demonstrated by inhibition of E-cadherin decrease, and N-cadherin or ZEB2 increase. These data suggest that CAFs promote the EMT of gastric cancer cells via the secretion of IL-6.

### CAFs-derived IL-6 mediates the migration and EMT of gastric cancer cells via the activation of JAK2/STAT3 pathway

**Figure 4:**
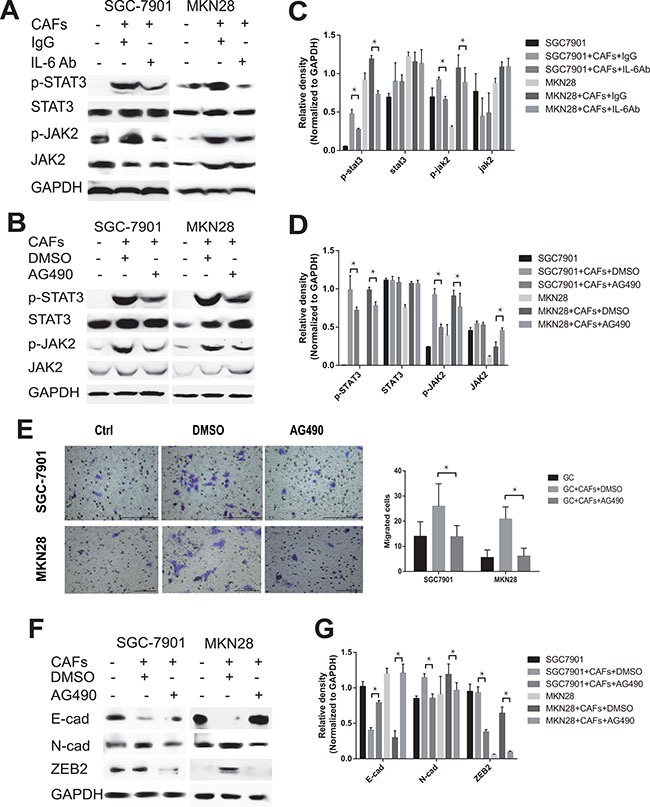
CAFs-derived IL-6 enhances the migration and EMT of gastric cancer cells via the activation of JAK2/STAT3 pathway (**A**, **B**) Protein levels of p-STAT3, STAT3, p-JAK2 and JAK2 in both SGC-7901 and MKN28 co-cultured with CAFs in the presence of IL-6 neutralizing antibody or AG490 were analyzed by western blot. Representative results from one of the three independent experiments are presented. (**C**, **D**) Densitometric analysis of p-STAT3, STAT3, p-JAK2 and JAK2 expression is shown. (**E**) The effect of CAFs on cell migration was assayed in the presence of AG490. Migratory Cells were counted in ten randomly selected microscopic fields. Values are represented as mean ± SD of three independent experiments. *P < 0.05. (**F**) Protein expression of E-cadherin, N-cadherin and ZEB2 in gastric cancer cells SGC-7901 and MKN28 co-cultured with CAFs in the presence of AG490 or equivalent concentration of DMSO was analyzed by western blot. Representative images from one of the three independent experiments are presented. (**G**) Densitometric analysis of E-cadherin, N-cadherin and ZEB2 expression is shown.

The canonical IL-6 signal transduction pathway is initiated by binding to IL-6R and phosphorylation STAT3 through the activation of JAK2. To interrogate the role of IL-6-JAK2-STAT3 pathway in mediating CAFs-induced migration and EMT changes of gastric cancer cells, we first explored the activation of IL-6-JAK2-STAT3 pathway in gastric cancer cells after co-culture with CAFs. As shown in Figure 4A–4D, CAFs significantly induced the phosphorylation of JAK2 and STAT3 in both SGC-7901 cells and MKN28 cells. In contrast, adding IL-6 neutralizing antibody or JAK-2 protein tyrosine kinase inhibitor AG490 into the co-culture system significantly reversed CAF-mediated phosphorylation of JAK2 and STAT3 in gastric cancer cells (Figure 4A–4D). We further examined whether block the JAK2/STAT3 pathway by AG490 could also inhibit the tumor-promoting effects on gastric cancer cells via the secretion of IL-6 by CAFs. As shown in Figure 4B and 4D, inhibition of JAK2-STAT3 pathway activation by AG490 significantly dampened cell migration (Figure 4E) and EMT (Figure 4F and 4G) induced by CAFs. These results indicate that IL-6-JAK2-STAT3 signal pathway plays an important role in CAFs-induced migration and EMT of gastric cancer cells.

### Blocking IL-6-JAK2-STAT3 pathway impairs the peritoneal dissemination and metastasis of gastric cancer cells induced by CAFs *in vivo*

**Figure 5:**
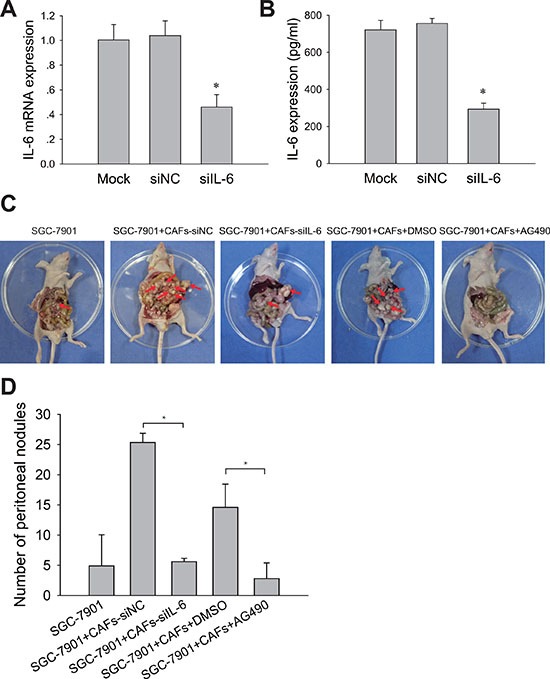
Blocking IL-6-JAK2-STAT3 pathway impairs tumor peritoneal dissemination and metastasis induced by CAFs *in vivo* (**A**) IL-6 mRNA levels in CAFs treated with siRNA or siNC were detected by qRT-PCR. (**B**) IL-6 protein concentration in the medium conditioned by CAFs treated with (siIL-6) or control siRNA (siNC) were measured by ELISA. (**C**) SGC-7901 cells and SGC-7901 cells mixed with CAFs that were transfected with IL-6 siRNA (siIL-6) or control siRNA (siNC) were inoculated into nude mice. Mice co-injected with SGC-7901 cells and CAFs were administered i. *p* with AG490 (500 μg/100 ul/mouse) or equivalent concentration of DMSO once a week. The peritoneal nodules (red arrows) were observed after 30 days (*N* = 5 per group). (**D**) Average peritoneal nodules from nude mice are shown. Data are representative of three independent experiments. *P < 0.05.

Peritoneal metastasis is a prevalent form of recurrence and metastasis in advanced gastric cancer. We further determined the contribution of IL-6-JAK2-STAT3 pathway to the peritoneal dissemination and metastasis of gastric cancer cells *in vivo*. CAFs were transiently transfected with human IL-6 siRNA (siIL-6) or with control scrambled siRNA (siNC). Knockdown of IL-6 expression in CAFs with IL-6 siRNA was confirmed by qRT-PCR and ELISA assay (Figure 5A and 5B). We then mixed CAFs with gastric cancer cells in a 1:4 ratio and intraperitoneally inoculated them into immunodeficient nude mice. As shown in Figure 5C and 5D, SGC-7901 cells mixed with CAFs-siIL-6 developed fewer peritoneal metastases than those developed by SGC-7901 cells mixed with CAFs-siNC (*P* < 0.05). We next examined whether inhibiting the activation of JAK2-STAT3 pathway by AG490 could also impair peritoneal metastasis induced by CAFs *in vivo*. We observed fewer peritoneal metastases of the CAFs/SGC-7901/AG490 group but not in CAFs/SGC-7901 group (Figure 5C and 5D). These results indicate that IL-6-JAK2-STAT3 pathway is required for peritoneal dissemination and metastasis of gastric cancer cells induced by CAFs *in vivo*, and pharmacologic blocking IL-6/JAK2/STAT3 signaling pathway in gastric cancer could reduce peritoneal dissemination and metastasis induced by CAFs.

## DISCUSSION

CAFs, the activated fibroblasts in cancer stroma, are the most abundant cells in the tumor microenvironment. Accumulating evidences demonstrate that CAFs play a prominent role in tumor growth and progression, and could be a promising tool to cancer therapeutics [26]. Therefore, a better understanding of the molecular mechanism for the tumor-promoting prosperities of CAFs is of obvious importance for understanding in gastric cancer progression and finding novel strategies to it. In this study, we demonstrate that IL-6 secreted by CAFs plays an important role in the progression of gastric cancer. We show that CAFs derived-IL-6 promoted the migration and EMT of gastric cancer cells via the activation of JAK2-STAT3 pathway, and blocking this pathway with IL-6 neutralizing antibody or JAK2 specific inhibitor AG490 impaired gastric cancer metastasis induced by CAFs *in vivo*. Therefore, the activation of JAK2-STAT3 pathway by IL-6 may play a central role in the interplay between CAFs and gastric cancer cells.

Previous studies have shown that cancer-associated stroma secretes a plethora of factors which promote the growth and invasion of the underlying tumor [16]. IL-6 is a multifunctional cytokine that was originally determined to be a regulator of immune and inflammatory responses [17]. Increasing evidences have suggested that IL-6 plays a critical role in modulating the function and activity of tumor-associated immune cells. For example, IL-6 inhibits the differentiation of dendritic cells, activates a Th17 cell response promoting tumorigenesis and expands myeloid derived suppressor cells (MDSC) [27]. In contrast to the effects of IL-6 on tumor-associated immune cells, IL-6 is also a direct critical driver of tumor growth and metastasis. Recent studies have shown that aberrant expression of IL-6 is tightly linked to tumor generation and poor disease outcome in many cancer types, including gastric cancer [18, 19, 28]. In this study, we reveal that IL-6 was overexpressed in both serum and cancer tissue of gastric cancer patients, and significantly higher expression was found in stromal fibroblasts. These results indicate CAFs is the major cell source in producing IL-6 in the tumor microenvironment of gastric cancer.

Interestingly, although NFs secreted lower IL-6 than CAFs, it also produced abundant IL-6 and significant higher than that from gastric cancer cells. Cancer-associated fibroblasts can be activated in incipient neoplasia to orchestrate tumor-promoting inflammation in an NF-kB-dependent manner [12, 29, 30]. Tissue-resident fibroblasts are suggested as precursors for CAFs, activated by tumor and immune cell-derived factors to express pro-inflammatory genes such as IL-1, IL-6, IL-8 and SDF-1. We previously showed that H. pylori infection induces the secretion of PGE2 by gastric epithelial cells that consequently silences stromal miR-149. The silence of miR-149 removes the suppression of its target gene IL-6, leading to elevated level of IL-6 that acts on fibroblasts to stimulate the transformation of NF into CAF [31]. Thus, cancer cells take advantage of the enormous plasticity of stromal fibroblasts and produce multiple signals that generate a tumor-promoting microenvironment.

EMT is an important embryonic process that is hijacked by tumor cells to facilitate execution of most of the invasion-metastasis cascade. Recently, Yadav *et al*. have shown that IL-6 induces EMT changes in head and neck tumor by up-regulating snail expression [32]. Breast cancer cells that constitutively expressed Twist, a EMT regulator and direct transcriptional repressor of E-cadherin, exhibited aberrant IL-6 production and STAT3 activation [33]. Initiation of metastasis requires tumor cell migration and invasion, which is enabled by EMT. In the present study, we have shown that IL-6 secreted by CAFs induced EMT of gastric cancer cells, which is characterized by losing epithelial markers E-cadherin and acquiring of mesenchymal markers N-cadherin and ZEB2. Then these EMT changes contribute to the enhanced capability of active locomotion of gastric cancer cells, which is demonstrated by increased migratory ability triggered by CAFs.

IL-6 exerts its effects by binding to a cell-surface type I cytokine receptor complex consisting IL-6a chain (CD126) and a common cytokine receptor signal-transducing subunit gp130, which forms a complex to activate STAT3 with the phosphorylation of Tyr705 via the JAK signaling pathway [20, 34]. Accumulating evidences show that activation of IL-6-JAK2-STAT3 signaling pathway by growth factors or cytokines plays an active role in tumor growth and progression. However, the role of CAFs and IL-6 in gastric cancer has not been well addressed. Our present study has shown that CAFs induced the phosphorylation of JAK2 and STAT3 of gastric cancer cells via the secretion of IL-6, and inhibiting JAK2-STAT3 pathway activation with AG490 significantly impaired cell migration and EMT, as well as peritoneal dissemination and metastasis *in vivo* induced by CAFs. CAFs are known to secrete multiple growth factors and chemokines such as SDF-1, VEGF, FGF, and CXCL14 into the tumor microenvironment that promote the growth and invasion of the underlying tumor by triggering multiple pathways [14, 35–37]. In the present study, we found that IL-6 neutralizating antibody partly suppressed the STAT3 or JAK2 phosphorylation, which suggested that IL-6 contributed partially to the tumor-promoting effect of CAFs on GC cells. Although we cannot preclude the likely involvement of other growth factors and/or cytokines, the studies of neutralizing IL-6 or inhibiting JAK2-STAT3 pathway activation with AG490 reveal that IL-6 is an important mediator in tumor-promoting effects of gastric CAFs that promotes EMT and peritoneal metastasis via the activating JAK2/STAT3 signaling pathway in gastric cancer.

In summary, we find that the crosstalk between gastric cancer cells and their stromal cells-CAFs contributes to tumor progression through IL-6-JAK2-STAT3 signaling. Recently, anti-IL6 directed therapies have been used clinically to treat various diseases, such as rheumatoid arthritis [38]. Thus, our results suggest that IL-6 targeted therapy could be a complementary approach against gastric cancer by exerting their action on stromal fibroblasts.

## MATERIALS AND METHODS

### Patient samples and cell lines

Plasma samples were collected from 66 preoperative gastric cancer patients, and 24 age-matched healthy volunteers. Gastric cancer tissues were obtained from 73 patients, between 2009 and 2012 at Ruijin hospital, School of Medicine, Shanghai Jiaotong University. None of the gastric cancer patients received radiotherapy or chemotherapy before surgery. Informed consent was obtained from each patient and healthy control for the use of their blood samples in this study. All the tissue samples were identified by clinical pathologist.

Gastric cancer cell lines SNU-1, MKN45, SGC7901 and MKN28 were purchased from Shanghai Institutes for Biological Sciences, Chinese Academy of Sciences, and were cultured at 37°C in a humidified atmosphere of 5% CO_2_ with RPMI-1640 medium containing 10% fetal calf serum with 100 U/ml penicillin and 100 U/ml streptomycin. Fibroblasts were isolated from 4 independent gastric cancer patients during radical gastric resection as previous described [14].

### Enzyme-linked immunosorbent assay (ELISA)

The protein levels of IL-6 in plasma and tissue lysates were measured by an ELISA kit (R&D Systems, Minneapolis, MN, USA) according to the manufacturer's instructions.

### Immunofluorescence

Tissues were fixed in 10% neutralized formalin and embedded in paraffin blocks. Sections (4 μm) were dewaxed with xylene and rehydrated with gradient ethanol, and then gently rinsed with phosphate buffered saline (PBS) for three times. After blocking with normal nonimmune goat serum for 30 min, tissue sections were incubated with anti-α-smooth-muscle actin and anti-IL-6 antibodies (Abcam, Cambridge, USA) at 37°C for 2 h. After 5 times rinses with PBS, cells were stained with appropriate Alexa dye-conjugated secondary immune reagents, Alexa dye-conjugated phalloidin, and Hoechst 33342 (Invitrogen, Carlsbad, CA, USA). Negative control staining was performed by omission of the primary antibody.

### Quantitative real-time PCR (QRT-PCR)

Total RNA was extracted using Trizol reagent (Invitrogen, Carlsbad, CA, USA) following the manufacturer's manual. RNA (1 μg) was reverse transcribed into cDNA using Reverse Transcription system (Promega, Madison, WI, USA). QRT-PCR was performed to quantify IL-6 mRNA level with the SYBR Green PCR core Reagent kit (Applied Biosystems, Foster city, CA, USA). GAPDH was used as the endogenous reference. Data were analyzed by using the comparative Ct method. Specificity of resulting PCR products was confirmed by melting curves. The primers used in this assay were: IL-6: 5′-CGGTCCAGTTGCCTTCTCCC-3′ (upper) and 5′-GAGTGGCTGTCTGTGTGGGG-3′ (lower); GAPDH: 5′-GGACCTGACCTGCCGTCTAG-3′ (upper) and 5′-GTAGCCCAGGATGCCCTTGA-3′ (lower).

### Cell migration assay

Cell migration assays were performed by using 8 μm transwell chambers (Corning Life Science, MA, USA). Cells were incubated in serum-free medium for 24 h and then were added to the upper chamber, and 5 × 10^4^ CAFs in 500 μl in RPMI 1640 medium containing 10% FBS were added to the lower chamber. Non-migrating cells from the interior of the inserts were removed with cotton-tipped swabs 48 h later, and cells that migrated to the bottom of the membranes were stained with 0.1% crystal violet for 30 min. The stained cells were counted and photographed. At least ten randomly selected fields were counted and the average number was presented.

### Western blot analysis

Cells were lysed with RIPA cell lysis buffer in the presence of protease inhibitor cocktail (Sigma, USA). The same amount of protein samples were loaded onto 10% SDS-PAGE and then transferred onto PVDF membranes. After blocked by skim milk, the membranes were incubated in the primary antibodies diluted by TBST buffer for overnight at 4°C and then in the HRP-conjugated secondary antibody for 2 h at room temperature. Finally the protein bands images were captured by a Tanon detection system with ECL reagent (Thermo). The primary antibodies used in the experiments were Anti-GADPH, anti-E-cadherin, anti-N-cadherin, and anti-ZEB2 antibodies were purchased from Proteintech.

### RNA interference

Human IL-6 siRNA and scrambled siRNA (Santa Cruz, CA, USA) at the final concentration of 100 nmol/L were transfected into CAFs cells with Lipofectamine 2000 reagent (Invitrogen, Carlsbad, CA, USA). Cells were collected for further assay at 24 h and 48 h after transfection.

### Tumor xenograft model and tumorigenicity assay

Male BALB/c nu/nu nude mice at the age of 4–5 weeks (Institute of Zoology Chinese Academy of Sciences), were housed at a specific pathogen-free environment in the Animal Laboratory Unit, Shanghai Jiao Tong University School of Medicine, China. Fibroblasts and SGC-7901cells were mixed at the ratio of 1:4 within 0.25 ml PBS and inoculated peritoneally. Some mice were administered i.p with AG490 (500 ug/100 ul/mouse) or equivalent concentration of DMSO once a week. All mice were sacrificed after 30 days and peritoneal metastasis nodules were counted. All animal studies were conducted with the approval of the Committee on Animal Care in Shanghai Jiao Tong University School of Medicine.

### Statistical analysis

Results were summarized as means ± SD. Student *t* test and one-way analysis of variance (ANOVA) was used to analyze the data and the significance level was set at *P* < 0.05.

## SUPPLEMENTARY MATERIALS FIGURES


