# Supplementary material for: IL-6 secreted by cancer-associated fibroblasts promotes epithelial-mesenchymal transition and metastasis of gastric cancer via JAK2/STAT3 signaling pathway
Source: Oncotarget. 2017 Feb 6;8(13):20741–50. doi: 10.18632/oncotarget.15119 (PMC5400541; doi:10.18632/oncotarget.15119)
Supplement: Supplementary file 1 [file oncotarget-08-20741-s001.pdf]

IL-6 secreted by cancer-associated fibroblasts promotes epithelial-mesenchymal transition and metastasis of gastric cancer via JAK2/STAT3 signaling pathway

Supplementary Materials

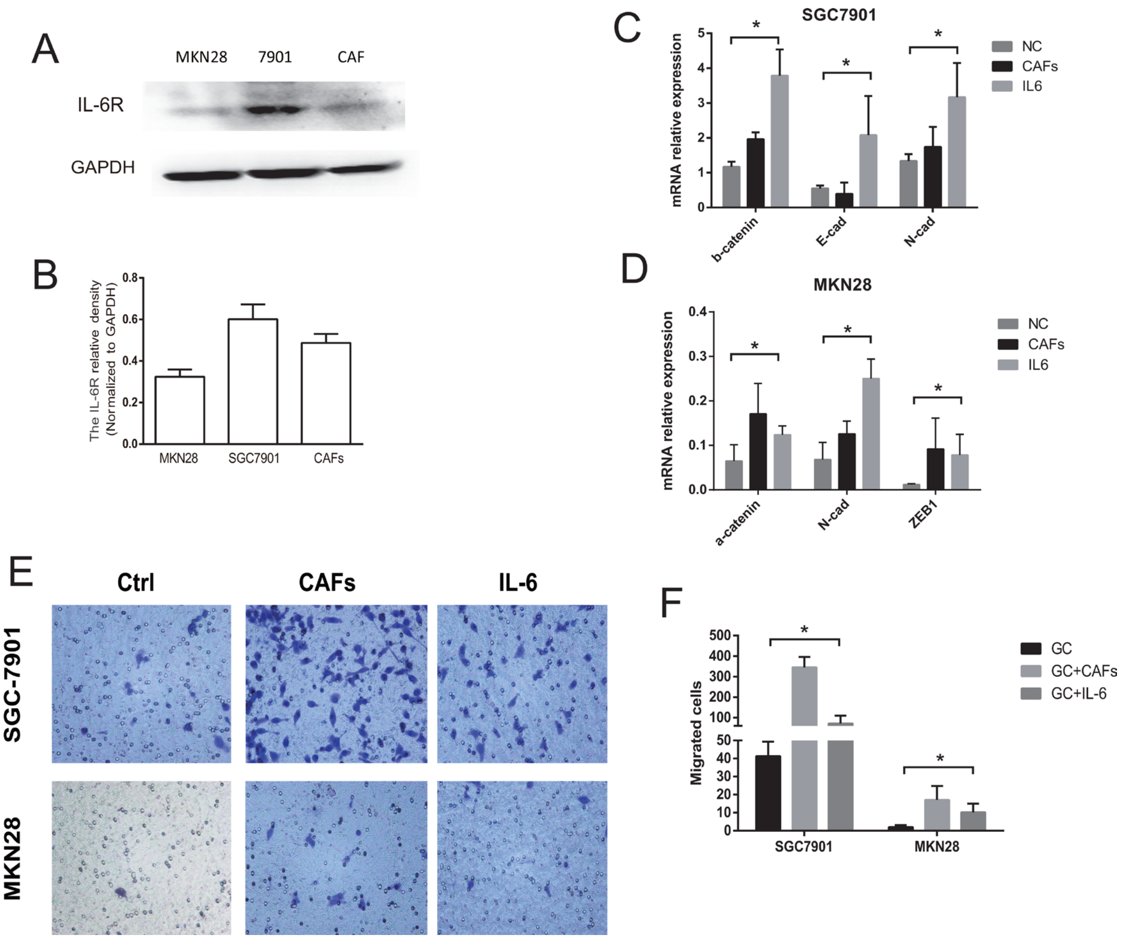

Supplement Figure 1: Exogenous IL-6 promotes EMT and migration of gastric cancer cells. (**A**) Protein levels of IL-6 receptor (IL-6R) in SGC-7901, MKN28 and CAFs were analyzed by western blot. Representative results from one of the three independent experiments are presented. (**B**) Densitometric analysis of IL-6R expression is shown. (**C**, **D**) The expression of EMT makers in SGC-7901 and MKN28 treated with recombined IL-6 or co-culture with CAFs were detected by qRT-PCR. (**E**) The effect of CAFs or IL-6 on cell migration was determined after 24 hrs. Representative photographs of migratory cells on the membrane (magnification, 100×) are shown. (**F**) Migratory Cells were counted in ten randomly selected microscopic fields. Values are represented as mean ± SD of three independent experiments. $^*P < 0.05$.
